# mSphere of Influence: Fungal behavior as a framework for the evolution of emergent traits

**DOI:** 10.1128/msphere.00651-24

**Published:** 2025-02-21

**Authors:** Andrew J. M. Swafford

**Affiliations:** 1 Department of Biology, Middlebury College, Middlebury, Vermont, USA; NC State University, Raleigh, North Carolina, USA

**Keywords:** chytrid, fungus, evolution, sensing, behavior

## Abstract

Andrew Swafford works in the field of evolutionary cell biology. In this mSphere of Influence article, he reflects on how the simultaneous introduction to the evolution of vision, the sensory biology of chytrid fungi, and a classic paper by Vrba and Gould helped shape his thinking about sensory evolution.

## COMMENTARY

I was initially drawn to evolutionary biology by my fascination with the intertwined evolutionary histories of emergent characters and their component parts. But defining complexity in these characters is a particularly difficult task, and unraveling their evolutionary history even more so. Just as behaviors can emerge from the coordination of sensory and motor pathways, a hallmark of emergent traits is that they arise when pathways, networks, or genes from different levels of biological organization become tightly integrated. I was first introduced to this concept in the classic paper “The Hierarchical Expansion of Sorting and Selection: Sorting and Selection Cannot Be Equated” by Vrba and Gould (1), and more recent publications on visual system evolution and fungal behaviors helped shape my conceptual framework around evolution, experimentation, and ultimately led me to an understudied group of fungi with surprising diversity in their sensory and motor systems.

In their 1986 publication in *Paleobiology*, Vrba and Gould argue that both sorting and selection lead to evolution but are differentiated by their causative process relative to the level of biological hierarchy being studied (e.g., genes, pathways, organisms, or species). Sorting is driven by interactions between levels or through causes outside of the focal level, with selection being only one such cause. They follow by introducing the roles of sorting and selection into three broad categories and provide several biological examples of how sorting and selection at one hierarchical level has impacted the evolutionary trajectory of others. One such example cites how selection at an organismal level favoring a gene conferring malaria resistance may have also driven a rise in an allele causing protan color blindness. A second example cites the hybrid sterility in *Clarkia* as an illustration of how emerging characters at the population level (e.g., small population sizes) can create sorting effects that fix mutations against the force of selection at the genomic level. Vrba and Gould ultimately posit that these complex evolutionary relationships emerge as previously independent pathways are integrated, resulting in an emergent character whose own evolutionary trajectory is entangled with that of its component parts. I found these hypotheses of dynamic relationships between evolution and complexity particularly compelling although Vrba and Gould had neither the data nor tools, at the time, to test them empirically. Doing so would require genomic data from hundreds of species alongside functional experiments in a broadly distributed, homologous, emergent trait that spans several experimentally tractable levels of biological organization.

Modern scientific advances have now bridged many of the gaps in knowledge identified by Vrba and Gould, bringing their hypotheses tantalizingly within reach. Thousands of genomes are now publicly available, bioinformatic tools now allow for structural predictions of similarity more in line with Vrba and Gould’s sorting hypothesis, and highly refined molecular tools now allow us to pick apart the component genes, proteins, and pathways underlying emergent traits. However, these advances have been developed sporadically across the tree of life, leaving important gaps in knowledge or methodology in currently tractable systems. Work on visual protein and pathway evolution (2–7) highlighted to me how sensory systems span multiple levels of organization—frequently bonding to other cellular processes to produce emergent traits like behavior. I was also introduced to chytrid fungi through reading about their unicellular, motile, and charismatic life stage, the zoospore. Elegantly, simple experiments scattered between the 1950s and late 1990s revealed that zoospores swim, crawl, and have integrated sensory systems with their motility, giving rise to behaviors that can guide recruitment onto suitable substrates for growth (8–10). More recently, existing and emerging diseases caused by chytrids (11, 12) intensified my interest in understanding the role chytrid sensory pathways play in their ability to identify and infect hosts. My simultaneous exposure to Vrba and Gould’s paper and the typically disparate fields of visual system evolution and fungal cell biology highlighted to me how developing our understanding of chytrid sensory systems and behaviors could strike the necessary balance between (relative) organismal ‘simplicity’ and sensory complexity in a diverse and ecologically important group of organisms.

The ideas in these papers have shaped my research trajectory into evolutionary cell biology, investigating the evolution of sensory systems and chytrid cell biology in order to build chytrids into a model system for testing evolutionary hypotheses. In my graduate research, I focused on chytrid chemosensory and visual systems and their integration into behavior (13) and delved into the evolutionary processes that might drive the evolution of emergent traits, like eyes and vision, from the integration of previously independent pathways (14, 15). In my postdoctoral work, I joined the effort to develop chytrids into a model system, which has seen incredible progress in molecular tools (16–18), chytrid cell biology (19–21), and sensory systems (13, 22, 23). I am excited to continue developing chytrids as a model system of sensory biology, leveraging this system to understand how the integration of sensory pathways, motility, and existing cellular processes can contribute to the emergence of pathogenicity and evolution of virulence. As I work toward answering the questions that first drew me into evolutionary biology, I am constantly reminded of the ways in which my research program builds upon the framework I was introduced to by Vrba and Gould.
